# Phylogenetic Analysis and Pathogenicity Assessment of the Emerging Recombinant Subgroup K of Avian Leukosis Virus in South China

**DOI:** 10.3390/v10040194

**Published:** 2018-04-13

**Authors:** Zijun Zhao, Mingzhang Rao, Ming Liao, Weisheng Cao

**Affiliations:** 1 College of Veterinary Medicine, South China Agricultural University, Guangzhou 510642, China; zzj2017825@163.com (Z.Z.); raomingzh@163.com (M.R.); mliao@scau.edu.cn (M.L.); 2 Key Laboratory of Veterinary Vaccine Innovation of the Ministry of Agriculture, Guangzhou 510642, China; 3 South China Collaborative Innovation Center for Prevention and Control of Poultry Infectious Diseases and Safety of Poultry Products, Guangzhou 510642, China; 4 National and Regional Joint Engineering Laboratory for Medicament of Zoonosis Prevention and Control, Guangzhou 510642, China; 5 Key Laboratory of Zoonosis Prevention and Control of Guangdong Province, Guangzhou 510642, China

**Keywords:** avian leukosis virus subgroup K, complete genome, phylogenetic analysis, pathogenicity assessment

## Abstract

In recent years, cases of avian leukosis virus (ALV) infection have become more frequent in China. We isolated 6 ALV strains from yellow feather broiler breeders in south China from 2014 to 2016. Their full genomes were sequenced, compared, and analyzed with other reference strains of ALV. The complete genomic nucleotide sequences of GD150509, GD160403, GD160607, GDFX0601, and GDFX0602 were 7482 bp in length, whereas GDFX0603 was 7480 bp. They shared 99.7% to 99.8% identity with each other. Homology analysis showed that the *gag*, *pol*, long terminal repeats (LTRs), and the transmembrane region (gp37) of the *env* genes of the 6 viruses were well conserved to endogenous counterpart sequences (>97.8%). However, the gp85 genes displayed high variability with any known chicken ALV strains. Growth kinetics of DF-1 cells infected with the isolated ALV showed viral titers that were lower than those infected with the GD13 (ALV-A), CD08 (ALV-B), and CHN06 (ALV-J) on day 7 post-infection. The infected Specific-pathogen-free (SPF) chickens could produce continuous viremia, atrophy of immune organs, growth retardation and no tumors were observed. These subgroup ALVs are unique and may be common in south China. The results suggested that updating the control and eradication program of exogenous ALV for yellow feather broiler breeders in south China needs to be considered because of the emergence of the new subgroup viruses.

## 1. Introduction

Avian leukosis virus (ALV), a member of family *Retroviridae* and genus *Alpharetrovirus*, is a common avian retrovirus associated with neoplastic diseases and other syndromes [1]. Based on the differences in host range, cross-neutralization patterns, and viral envelope interference, ALVs isolated from chickens can be divided into 6 subgroups (A, B, C, D, E, and J). According to the characteristics and mode of transmission, subgroups A, B, C, D, and J are considered as exogenous viruses, whereas subgroup E encompasses the endogenous viruses [2,3]. Viruses of subgroups A and B are common pathogenic exogenous viruses in the field [4], while the viruses of subgroups C and D have rarely been reported [5]. Subgroup E, which includes the ubiquitous endogenous leukosis viruses, has little or no oncogenicity, but can interfere in the detection results of exogenous ALV and hence cause great difficulties in the eradication of exogenous ALV [6]. The subgroup J virus was first isolated from meat-type chickens in 1989 in the United Kingdom. This virus chiefly induces myelocytomatosis and has caused enormous economic losses to breeding companies worldwide [7].

The gp85 gene encodes the most variable of the structural proteins in the genome of ALV, which is associated with virus neutralization and viral host range [8,9]. This genetic variability of the gp85 gene allows for the development of new or recombinant retroviral strains that escape host defenses, present immune tolerance, or resist antiviral drugs. In recent decades, different subgroups of ALV strains have been isolated worldwide. In 1991, Payne [3] described ALV-J, a new isolate of ALV from meat-type chickens. A clinical case of tumors caused by ALV-J infection was firstly reported in egg-type commercial layers in 2004 [10]. Furthermore, ALV-J infections have also been reported in several Chinese local chicken lines, indicating that the pathogenicity of ALV-J may have enhanced considerably, which in turn might have expanded its host range [11,12]. In 2012, the following 3 ALV strains were isolated from the Chinese native chicken breed “luhua:” JS11C1, JS11C2, and JS11C3. These isolates were suggested to be part of new subgroup Kdue to relatively distant relations to any of the known subgroups in their gp85 gene [13,14]. Similarly, a unique ALV strain named TW-3593 was reported in Taiwan [15] and several fowl glioma viruses (FGV) isolated from Japan also showed low similarity compared to the other ALVs in their gp85 sequences [16,17,18,19,20]. Hence, these isolates were identified as a new subgroup. TW-3539 was unique because the long terminal repeat (LTR) of this isolate displayed high identity to the endogenous counterpart sequence and its gp85 was different from all chicken subgroups of ALVs.

In recent years, cases of ALV infections have become more frequent in China. The control and eradication of ALV has become a major challenge in the poultry industry because of inefficient organization and unavailability of effective drugs or vaccines [21]. The yellow feather broiler breeders have also been facing serious problems due to ALV infections [22]. In the present study, 6 ALV strains were isolated from yellow feather broiler breeders in the Guangdong Province from 2014 to 2016. To gain a better understanding of the evolutionary relationship and the molecular characteristics of the isolates, their complete genome sequences were amplified, sequenced, compared, and analyzed with other reference strains of ALV. Phylogenetic analysis of their gp85 genes with other known strains of subgroups A, B, C, D, E, and J revealed that the 6 viruses and ALV subgroup K strains JS11C1and JS13-DX5 were all included in the same cluster, which is different from all 6 known subgroups of ALVs, suggesting that they belong to a new subgroup-subgroup K (ALV-K). Therefore, it is necessary to further study its biological characteristics, genome structure and function, and the molecular mechanism of pathogenicity. This study reported 6 novel ALV-K isolates from yellow feather broiler chickens in south China. These K-like ALVs were unique. The LTR of these isolates displayed high similarity with the endogenous counterpart sequence and their gp85 was different from all chicken subgroups of ALVs. This study is expected to contribute to our understanding on the control and eradication program of exogenous ALV for yellow feather breeders in the Guangdong Province, China.

## 2. Materials and Methods

### 2.1. Samples and Cells

A total of 254 anticoagulation blood samples were aseptically collected from the apparently healthy roosters of 3 different flocks of yellow feather broiler breeders under the exogenous eradication program in the Guangdong Province of China, of which 169 were collected in 2014, 35 were collected in 2015, and 50 were collected in 2016. The sampled roosters had no reported clinical signs of tumors. The plasma homogenates were aseptically separated after centrifugation at 2000× *g* for 10 min at 4 °C and stored at −80 °C for virus isolation. The DF-1 fibroblastic cell line (American Type Culture Collection, Manassas, VA, USA) was kept in the Key Laboratory of Veterinary Vaccine Innovation of the Ministry of Agriculture, Guangzhou, China.

### 2.2. Virus Isolation

All plasma samples were examined by inoculating into DF-1 cells, which are known to be susceptible only to exogenous ALVs [23]. The procedures for the isolation and identification of ALV in cell cultures were performed as previously described [24]. Briefly, plasma homogenates were inoculated into DF-1 cell monolayer and cultured in Dulbecco’s modified Eagle’s medium (DMEM, Invitrogen, Shanghai, China) supplemented with 10% fetal bovine serum (FBS, GIBCO) for 2 h at 37 °C in a 5% CO_2_ incubator (Thermo Fisher Scientific, Waltham, MA, USA). The cell culture medium was discarded and replaced with fresh DMEM containing 1% FBS, 100 U/mL penicillin, and 100 µg/mL streptomycin. The infected cells were maintained at 37 °C with 5% CO_2_ for 7 days with daily monitoring. The supernatant of the cell culture was tested for ALV group-specific antigen (p27) by antigen-capture enzyme-linked immunosorbent assay (ELISA) (IDEXX Laboratories, Westbrook, ME, USA) according to the manufacturer’s instructions. ELISA-positive samples were harvested for DNA extraction and polymerase chain reaction (PCR). Genomic DNA from uninfected DF-1 cells was used as a negative control.

### 2.3. Extraction of Proviral DNA and Primers

The proviral genomic DNA samples were extracted from ALV p27 antigen positive DF-1 cells. To amplify and obtain the full-length proviral genome of the isolates, 4 specific primers (Table 1) were designed and synthesized based on the published nucleotide sequence of TW3593 [15].

### 2.4. Genomic DNA Amplification and Sequencing

Total genomic DNA from infected DF-1 cells was used as a template for PCR amplification. PCR was performed in a 50 µL reaction mixture that consisted of template DNA (5 µL), 10× PCR buffer (TaKaRa, Dalian, China), 1 µM of forward and reverse primers, 2 mM of MgCl_2_, 100 mM of each dNTP, and 1 unit of LA Taq^TM^ DNA Polymerase (TaKaRa, Dalian, China). PCR products were analyzed on 1% agarose-Tris-acetate-EDTA (TAE) gels, stained with ethidium bromide and photographed using a gel documentation system (UVITEC, Cambridge, UK). The gel-purified PCR products were cloned into pMD18-T vector (Takara, Dalian, China) and transformed into DH5α *Escherichia coli* competent cells (Takara, Dalian, China). Clones containing recombinant plasmids were confirmed by PCR. DNA sequences from the positive clones were determined by Biotechnology Company (Invitrogen, Shanghai, China), and 3 independent recombinant plasmids were sequenced to confirm the accuracy of the sequences.

### 2.5. Sequence Analysis

The complete genome sequence alignments were compared with other ALV reference strains published in the National Centre for Biotechnology Information GenBank database. The nucleotide and deduced amino acid sequences were aligned in the sequence analysis software Lasergene (version 7.10) by using the Clustal W method in the MegAlign program (DNASTAR, Madison, WI, USA). Phylogenetic analysis of complete proviral sequences of the ALV isolates was performed with the neighbor-joining and maximum parsimony methods, with 1000 bootstrap replicates, using MEGA ver.5.05 [25]. The GenBank accession numbers of strains used in this study are listed in Table 2.

### 2.6. Evaluation ofthe Replication Ability of ALV-KIsolates In Vitro

0.1 mL of ALV-A (GD13), ALV-B (CD08), ALV-J (CHN06), and ALV-K (GDFX0601) at a concentration of 10^3.2^ 50% Tissue culture Infective Dose (TCID_50_) mL^−1^ was inoculated per well in 24-well cell culture plates containing 1 mL 1.7 × 10^5^ cells/well DF-1 cells in triplicate. Three wells on each plate served as the negative control. The infections were carried out in the presence of 1% FBS at 37 °C under 5% CO_2_ and harvested on day 7 post-infection. After 3 freeze-thaw cycles, the harvested samples were examined for ALV group-specific p27 antigen (ELISA) to determine the replication ability. Each sample was tested independently 3 times.

### 2.7. Animal Experiments

A total of 110 1-day-old specific pathogen free (SPF) chickens (white leghorn; SAIS poultry Co., Ltd., Jinan, China) were randomly divided into 3 sterilized isolators (Group I: 30 chickens; Group II: 40 chickens; and Group III: 40 chickens). Chickens in groups II and III were injected subcutaneously with 0.2 mL and 0.4 mL of 10^4^ TCID_50_ of ALV-K (GDFX0601), respectively. Each chicken in group I served as a normal control, and was not subjected to any treatment. The treated chickens were examined daily for symptoms of illness and anti-coagulated blood samples. The cloacal swabs were collected on days 7, 14, 21, 28, 35, 65, 95, and 125for viremia detection and ALV p27 antigen testing. On days 7, 14, 21, 28, 35, 65, 95, and 125, 5 chickens from each group were selected randomly and euthanized to determine the immune organ weight, and viral load in organs was tested on 125 days post infection. Organs, including the liver, spleen, kidney, heart, lung, stomach, cecal tonsil, pancreas, and bursa were collected. The spleen and bursa were excised and weighed to determine indices of the immune organs, expressed as the weight of immune organs to live body weight. Feeding of the chickens was stopped 12 h before sampling. The chickens were monitored for 125 days after virus treatment and euthanized at the end of the experiments. The real-time RT-PCR with primers designed for the envelope gene and gene-specific primers: F: 5′-CCCCTGCTATTTAGGCAAGCT-3′, R: 5′-AGTTGGCAAGCACCTTGAGAA-3′, Probe: Fam-5′-CCATGTTAGCACCCAACCACACAGAA-3′-Eclips synthesized by TaKaRa Company (Dalian, China).

### 2.8. Ethics Approval

Our animal research was conducted under the guidance of the SCAU’s Institutional Animal Care and Use Committee. The chicken sampling procedures were approved by the Animal Care and Use Committee of Guangdong Province, China (2016-B024, April, 2016).

## 3. Results

### 3.1. Virus Isolation

All the plasma samples cultured in DF-1 cells were detected with ALV p27 antigen ELISA. The p27 antigen positive rate in those flocks were 1.78% (3/169), 3.57% (2/56), and 3.45% (1/29) indicating that the rooster flock of yellow feather broiler breeders experienced an exogenous ALV infection. Six ALV strains were isolated from the plasma samples and named as GDFX0601, GDFX0602, GDFX0603, GD150509, GD160403, and GD160607.

### 3.2. Amplification of Proviral Genomes

**Figure 1:**
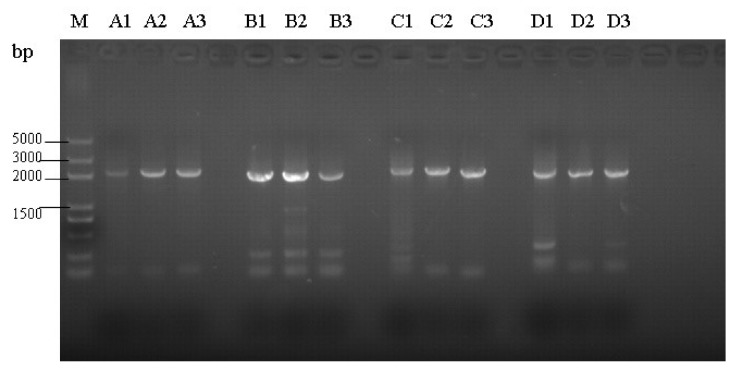
PCR amplification of the complete genomes of 3 isolates. M: DNA marker DL5000; 1–3: GDFX0601, GDFX0602, and GDFX0603; A, B, C, D: 4 DNA fragments of the complete genome.

**Figure 2:**
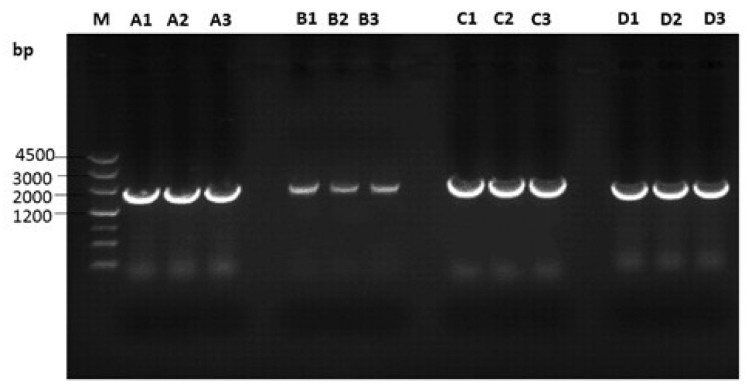
PCR amplification of the complete genomes of 3 isolates. M: DNA marker 3; 1–3: GD150509, GD160403, and GD160607; A, B, C, D: 4 DNA fragments of the complete genome.

The 6 ALV isolates from DF-1 cells inoculated with plasma were subjected to full proviral genome sequencing. Genomic DNA from infected DF-1 cells was used as a PCR template, and 4 DNA fragments A, B, C, and D with expected lengths of about 2147, 1989, 2196, and 1993 bp, respectively, were amplified (Figure 1 and Figure 2).

### 3.3. Complete Genome Sequences Analysis of ALV Isolates

**Figure 3:**
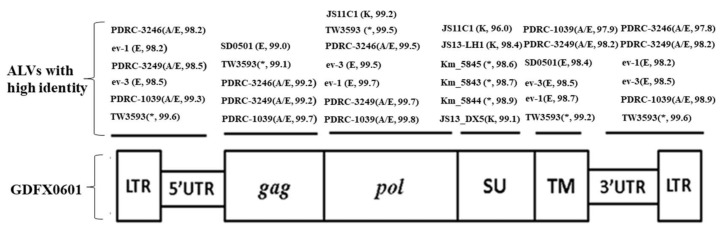
The genome structure of GDFX0601. The reference strains with high sequence similarity to this isolate are shown above the genome structure. *: The isolates whose subgroups are not known.

The full-length proviral genomes ofGDFX0601, GDFX0602, GD150509, GD160403, and GD160607 were 7482 nucleotides in length, whereas GDFX0603 was 7480 nucleotides long. The differences in genome length among the isolates are caused by a base deletion in the 5′LTR region and 3′UTR region of the GDFX0603 isolate. They shared 99.7% to 99.8% genome sequence identity with each other. All the isolates displayed a genetic organization typical of replication-competent genus Alpha retrovirus lacking viral oncogenes (Figure 3), which indicates that the isolates would be chronically transforming ALVs. The 3 main genes of the 6 ALV isolates are *gag*, *pol*, and *env*, and they are 2106 bp, 2688 bp, and 1791 bp in length, respectively. LTRs located at 5′ and 3′ ends of the isolates were both 274 bp in length, including the U3-R-U5 element whereas GDFX0603lacked a base at 5′LTR.

The sequence identities of the major structural genes with the ALV reference strains showed that the *gag* and *pol* genes of the 6 isolates were well conserved with all other ALVs, sharing 95.3–99.7% and 97.2–99.8% nucleotide similarities, respectively. All the 6 isolates exhibited very similar sequences to the ev-1 locus with at least 98.6% identity for the *gag* gene and up to 99.8% for the *pol* gene (Figure 3). However, the *env* genes were much more variable, sharing 57.1–97.7% identity with other reference strains.

**Figure 4:**
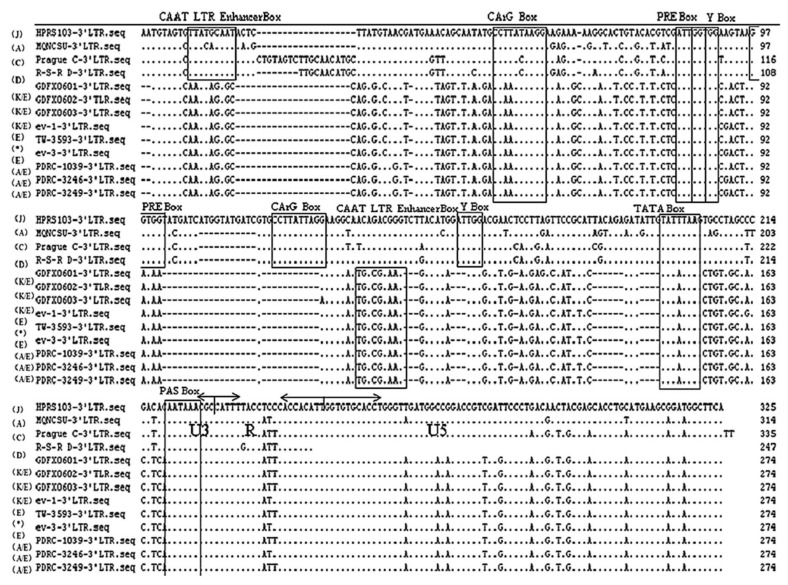
Nucleotide sequence alignments and motifs of the ALV LTR region. Dots (·) indicate identical residues, while letters indicate base substitutions. Dashes (–) indicate gaps in the alignment. Locations of putative transcriptional regulatory elements are indicated in boxes and are marked.

Homology analysis of the proviral LTRs showed low similarity with those of exogenous ALVs, represented by <70.3% nucleotide similarity compared with ALV-A, ALV-B, ALV-C, ALV-D, and ALV-J (Table 3). However, the LTRs of all isolates were highly similar to endogenous loci, represented by at least 96.5% similarity with SD0501, ev-1, and ev-3, and as high as 97.8% to 100% similarity with TW3593, PDRC-1039, PDRC-3246, and PDRC-3249 (Table 3). The R regions within the LTRs of the isolates showed little divergence compared with all the reference ALVs, sharing 100% similarity with TW3593, PDRC-1039, PDRC-3249, Prague C, JS11C1, and representing 81–95.2% identity to other reference strains. U5 regions were 100% similar to the ev-1 loci in all isolates and showed only 83.3% to 93.6% similarity with exogenous viruses. In addition, transcriptional regulation elements of the 3 isolates were identified in the U3 region including CArG box, Y box, PRE box, CAAT box, and TATA box (Figure 4). Almost all of the putative transcription regulatory elements identified in the U3 region were conserved and homologous to those of USA isolates PDRC-1039, PDRC-3246, PDRC-3249, and TW3593 isolate and the endogenous viruses.

The gp85 (SU) and gp37 (TM) coding regions in the envelope gene of the 6 ALV isolates were much more variable. The gp37 genes exhibited only 58.7–59.7% similarity with the ALV-J reference strains, and 95.7–99.5% similarity with several ALVs isolated from indigenous chicken breeds in the Chinese mainland (JS11C1), Taiwan region (TW3593), Japan (Km_5844, Km_5845, Km_5943, etc.), and USA (PDRC-1039, PDRC-3246, PDRC-3249) in recent years (Table 3). Homology analysis of the gp85 genes showed only 51.5–88.5%similarity compared to the homologous sequences of ALV subgroups A, B, C, D, E, J; whereas as high as 94.5–99.2% similarity with the ALVs isolated from China mainland (JS11C1, JS13-DX5, JS13-LH1), Taiwan region (TW3593), and Japan (Km_5844, Km_5845, Sp_53, etc.) (Table 3 and Figure 3) was detected.

### 3.4. Phylogenetic Analysis of 6 ALV Isolates

**Figure 5:**
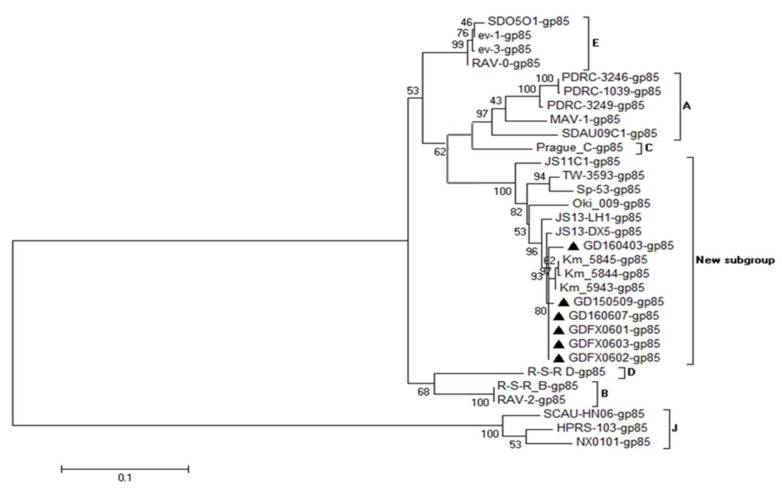
Phylogenetic relationship of the 6 novel isolates to reference ALV strains, based on the gp85 genome sequences. Black triangle (▲) indicates ALV isolates in this study.

Phylogenetic analysis of the gp85 gene sequences of the 6 subgroup K-like ALV isolates and other ALV reference strains demonstrated that all the 6 above isolates, TW3593, JS11C1, JS13-DX5, JS13-LH1, and several Japanese isolates were clustered in one big clade, which was different from all 6 known ALV subgroups from chickens (Figure 5), indicating that the 6 isolates might be classified as a new subgroup (subgroup K). However, the *gag*, *pol*, and LTR genes of the 6 isolates were well conserved with endogenous ALVs, revealing that the ancestor of 6 isolates probably emerged by the recombination of ALV-K like viruses with some endogenous ALV-E viruses.

### 3.5. Evaluation ofthe Replication Ability of Subgroup K ALV Isolates in DF-1 Cells

**Figure 6:**
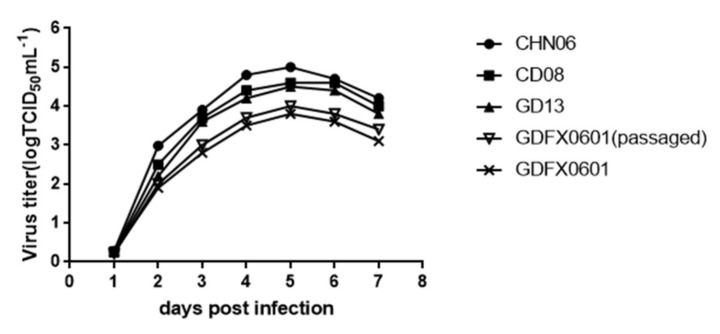
Replication of the GD13 (ALV-A), CD08 (ALV-B), CHN06 (ALV-J), and GDFX0601 in DF-1 cells.

The influence of the ALV-E like LTRs on ALV-K replication was evaluated in vitro using DF-1 cells, which are commonly used as host cells for ALV proliferation. DF-1 cells were infected with GD13 (ALV-A), CD08 (ALV-B), CHN06 (ALV-J), and GDFX0601 (ALV-K). Culture supernatants of cells infected with the GD13, CD08, and CHN06 strains had relatively higher viral titers on day 7 post infection than those infected with E-like LTRs ALV-K (Figure 6). DF-1cells were infected with ALV-K and passaged continuously for 3 more generations so that virus titers would rise. These results showed that the ALV-K isolate replicated more slowly than the GD13 (ALV-A), CD08 (ALV-B), and CHN06 (ALV-J) strains in DF-1 cells.

### 3.6. Influence of ALV-K Infection on Growth Rates of SPF Chickens

**Figure 7:**
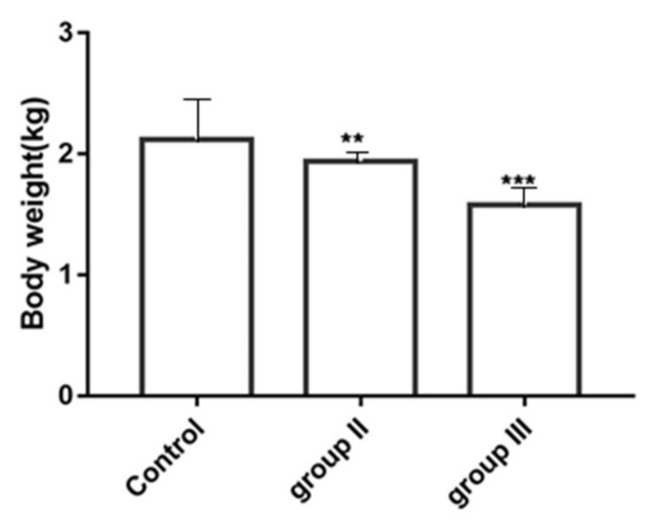
Influence of ALV-K infection on body weights in Specific-pathogen-free (SPF) chickens. Mean weight was significantly different between the groups (*p* < 0.01). ** *p* < 0.01, *** *p* < 0.001.

Body weights are summarized in Figure 7. Chickens inoculated with low dose ALV-K had significantly lower weights than control group chickens (*p* < 0.01). However, chickens inoculated with high dose ALV-K had significantly lower weights compared with chickens in all other groups (*p* < 0.001).

### 3.7. The Influence of ALV-K Infection on Mortality

The control group and low-dose group (200 µL 10^4^ TCID_50_) had 0% mortality rates, whereas the high dose group had mortality rates of 3%, 3.4%, and 3.6% on days 14, 21, and 28, respectively (Table 4). The result indicated that chickens infected with ALV-K have low mortality rates.

### 3.8. Influence of ALV-K Infection on Spleen and Bursa

**Figure 8:**
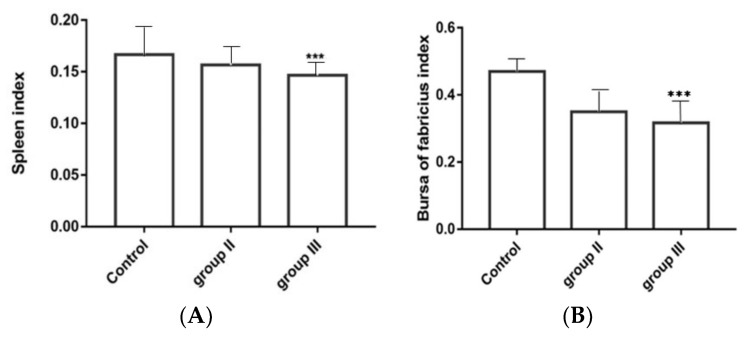
The spleen and bursa were excised and weighed to determine indices of the immune organs, expressed as the weight of immune organs to live body weight. (**A**) spleen index; (**B**) bursa index. *** *p* < 0.001.

The immune-organ to body weight in high dose ALV-K infected group was significantly lower compared with those in the control group (*p* < 0.001) (Figure 8A,B).The spleen index of the low dose group was lower than that of the control group, and the differences in bursa index between the low-dose group and the double-dose group were not significant.

### 3.9. Detection of Viremia and Cloacal Swabs ALV p27 Antigens

**Figure 9:**
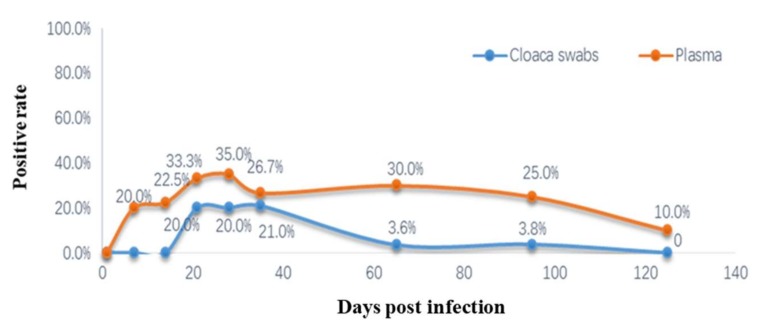
Ratios of samples positive for viremia from the infected groups as detected by plasma virus isolation and cloacal swabs ALV p27 antigens.

Cloaca swabs and whole-blood samples collected from the SPF chickens that were artificially inoculated with ALV-K were analyzed by virus isolation and ALV p27 antigen ELISA on days 7, 14, 21, 28, 35, 65, 95, and 125 post-infection. In vivo replication of ALV-K (GDFX0601) was measured by determining the viremia levels in chickens. The ALV-K was detected at 7 dpi from the whole-blood samples. However, ALV-K p27 antigen detected from the cloacal swabs showed no positivity on days 7 and 14. The positive ratio of viremia in the infected group reached 35% on day 28 post-inoculation. Thereafter, it declined and remained at a low level (<30%) for 125 days and never increased in subsequent samplings. The results of group II and group IIIis no difference. Another phenomenon of concern was that the ratio of ALV-positive cloacal swabs from the infected group remained at a low level (<21%) from days 21–35 post-inoculation, and few ALV-positive chickens were detected from the cloacal swabs of the infected group (group II and group III) at the end of the testing period (Figure 9). Notably, the data plasma detection rate was higher than that of the cloacal swabs. In the control group, no viruses were detected from both the cloacal swabs and the whole blood samples.

### 3.10. The Distribution of ALV-K in Different Organs at the Later Stage of Infection

**Figure 10:**
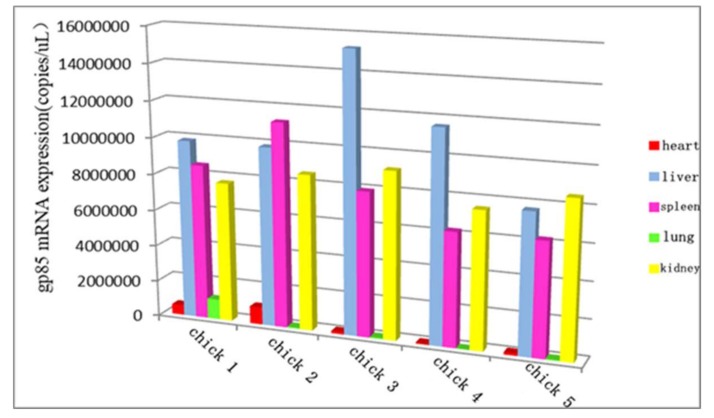
Real-time PCR method to investigate the distribution of ALV-K in different organs at the later stage of infection.

The routine PCR and real-time PCR methods were applied to investigate the distribution of ALV-K in different organs at the later stage of infection. The viral loads of the liver, spleen, and kidneys were higher than those of other organs when chickens were euthanized at 125 days of age, and ALV-K could be detected in the liver, spleen, kidney, heart, lung, and bursa (Figure 10). No tumors were observed in challenged birds when all the remaining infected chickens were euthanatized on day 125 post-infection.

## 4. Discussion

ALV is a member of RNA viruses, which contains the overall structure of a typical slow-transforming replication-competent ALV: 5’-LTR-leader-gag/pol-env-LTR-3’ [26,27]. Although ALV has been substantially purified completely in other countries, there are still reports of ALV infection in China due to scattered farms, the variety of local chicken species, and so on [28,29,30,31]. In 2012, three novel ALV strains (JS11C1, JS11C2, and JS11C3) were isolated from the Chinese native chicken breed “luhua”. Because of the low homology between their gp85 sequences and other known subgroups, they were named ALV-K [13,14]. ALV-K has increasingly been isolated from Chinese native chickens recently, but the specific phylogeny and pathogenicity of ALV-K is not yet clear.

In this study, the following 6 field ALV strains: GDFX0601, GDFX0602, GDFX0603, GD150509, GD160403, and GD160607 were isolated from apparently healthy yellow feather broiler breeders of 3 different flocks in Guangdong Province of China. In order to explore their phylogenetic profile, first of all, their entire genome structure was analyzed and compared with other classical strains of known ALV subgroups. All 6 isolates were extremely similar to each other at the nucleotide level (>99.7%), suggesting that they shared the same ancestor. Complete proviral genome sequence analysis indicated that the gag and pol genes of the 6 isolates were relatively conserved compared to those of other reference strains, showing >95.3% nucleotide similarity. All the 6 isolates exhibited very similar sequences to the endogenous ALVs, with a minimum of 98.6% similarity for the gag gene and up to 99.2% for the pol gene (Figure 3).

The gp85 genes of the 6 isolates were the most variable, with similarities to the reference subgroups A to E of <88.5%, and they shared only 51.7% similarity with ALV-J (Table 4). The phylogenetic analysis of the gp85-based phylogenetic tree containing 6 isolates also contained TW3593 [15] which was isolated from indigenous chickens in Taiwan in 2010. JS11C1, JS13-LH1, and JS13-DX5, are classified as subgroup K and were isolated in mainland China in 2012 [13] from indigenous chickens with liver tumor, hemangioma, emaciation, and egg production drop, while the other 5 strains (Km_5843, Km_5844, Km_5845, Oki_009, Sp_53) were all isolated from Japanese bantams identified by fowl glioma [17,18,19,20]. The results further prove that these 6 strains of ALV isolates belong to ALV-K. Although the six ALV strains are isolated from healthy flocks, the potential threat of ALV-K cannot be ignored because of the tumorigenic effects of other viruses in the same branch. Further studies are still needed to explore the factors that determine the pathogenicity of ALV-K and ALV-K-like viruses.

The 3′UTR of avian retroviruses is important in viral pathogenesis and replication [32], the LTR enclosed within the 3′UTR contains powerful transcription regulatory elements that might differ among viruses and determine the chromosomal and viral gene expression. According to sequence analysis of the putative transcription regulatory elements (including CArG, CAAT, and TATA boxes) [33] in the U3 region, the 6 isolates showed low identity to equivalent sequences of exogenous reference viruses and exhibited <67.0% similarity with the strains of ALV subgroups A, B, C, D, and J, and the similarity to the first isolated strain, JS11C1, is only 71%. In contrast, the U3 sequences were more similar to the endogenous viruses (Figure 2). The CCAAT enhancer box was absent in the 6 viruses and the endogenous viruses, butis present within the first 20 bp of U3 in exogenous viruses. However, at positions 104–113 of the 6 viruses and all endogenous viruses, the same consensus sequences (TT/GNNGC/TAAT/G) [34] were examined, which constitutes a functional enhancer box (Figure 3). Within the U3 region, only the first of two CArG boxes known to be present in exogenous viruses is present in the 6 ALVs, a feature that is characteristic of endogenous loci LTRs (Figure 3). The Y boxes (with sequence ATTGG, also known as inverted CCAAT boxes) were conserved in all the reference viruses [32], but the penta-nucleotide repeat element boxes (with sequence GGTGG) were absent in the 6 isolates and the endogenous viruses. The TATA box (TATT/ATAA consensus) and the polyadenylation signal (AATAAA) were well conserved in all the reference viruses (Figure 3). In addition, the R and U5 regions within 3′LTR also exhibited high similarity with the endogenous ALVs. The recombination of endogenous and exogenous strains demonstrated by these 6 isolates was identical to that of Taiwan isolate TW3593 [15], isolates from other parts of mainland China, and Japanese glioma isolates [17,18,19,20], but it was different from the first ALV-K isolate, JS11C1 [15]. Whether the subgroup virus itself is a recombination virus or it is formed under the advantage of superiority, it needs further verification through quasi-species analysis. Some scholars believe that ALV-J is also an endogenous and exogenous ALV recombination virus [16]. Therefore, taking into account the serious economic losses that ALV-J has brought to farms, the threat of this type of ALV-K should not be underestimated and its pathogenic mechanism needs to be further analyzed to formulate a corresponding purification plan.

Second, in order to assess their proliferation and pathogenicity, the replication kinetics of the cells infected by ALV-A/B/J/K should be compared in vitro and tested with the ALV p27 antigen ELISA. SPF chickens were infected with different volume of inoculum virus and the effect on body weight, immune organ index, viremia, and virus philophilicity were analyzed. The results showed that GDFX0601, GDFX0602, and GDFX0603 replicate more slowly in DF-1 cells than the GD13 (ALV-A), CD08 (ALV-B), and CHN06 (ALV-J) strains. This result is consistent with the viruses with endogenous ALV LTRs, which may be responsible for lower viral transcription and pathogenicity [23]. It may also be the reason why ALV-K is frequently isolated from healthy chickens. ALV-K may have existed in Chinese local chickens for a long time and probably as a persistent infection, due to its weak replication ability. ALV-K can easily escape detection and is vertically transmitted. Similarly, once infected, it is very difficult to eradicate. Therefore, ALV-K infection poses a serious threat to the economic viability of the involved flocks, their progeny, and the associated poultry industry.

Usually, ALV-K causes subclinical infections leading to growth retardation and immune organ atrophy that may cause immunosuppression, which is often neglected until clinical signs develop. In this article, ALV-K possessing endogenous ALV LTRs that infect SPF chickens do not induce tumor formation, however, they cause not only serious growth retardation, but also severe inhibition of the growth of immune organs and may also cause death of chickens. Spleen and bursa are the major immune organs of poultry and the weight of immune organs directly reflects their development. We found that the weight of immune organs relative to their body weight in double-dose infection was significantly lower than the weight in the control group (*p* < 0.001) at 35 dpi. In the low-dose infection group, only the weight of the bursa was lower than the control group, whereas in the double-dose group, both the spleen and bursa were much smaller. This suggests that ALV-K infection could possibly cause atrophy of immune organs of poultry. We also found the viral loads of the liver, spleen, and kidneys were higher than those of other organs. This result seems to be inconsistent with the clinical symptoms. A possible reason is differing levels of infection. However, the test results suggest that although ALV-K does not show subclinical symptoms, it must not be ignored.

In summary, we isolated 6 novel ALV-K ALVs from yellow feather broiler breeders in south China, which may further complicate the control and eradication program of ALV from yellow-feather chickens. We found that ALV-K isolates replicate more slowly and are less pathogenic than some other ALV strains. Our findings may help to reveal the evolution of ALV strains prevalent in fields, which may be beneficial for research on the pathogenicity and biological characteristics of novel ALV strains.

## Figures and Tables

**Table 1:** Primers used for amplifying the full-length proviral genome in this study.

Primer ^a^	Sequence (5′–3′) {Nucleotide Position of Primer}	Product Size (bp) {Nucleotide Position}
K-1F	TGTAGTCAAATAGAGCCAG {1–19}	2147
K-1R	GGCTGTTTCCTGACTTT {2131–2147}	{1–2147}
K-2F	GTCACAGCCAGATATTCAG {1858–1876}	1989
K-2R	AGGCAACAGGAGGATAT {3830–3846}	{1858–3846}
K-3F	AGCGTTAGGAATCCCGCCACGA {3468–3489}	2196
K-3R	TGGTGCTGTTGTCAGGGCCGC {5643–5663}	{3468–5663}
K-4F	TCTGCCTCTCTACACAGTCAGCCACCTCCC {5490–5519}	1993
K-4R	TGAAGCCTTCTGCTTCATTCAGGTGTTCGCAATC {7449–7482}	{5490–7482}

^a^ F and R represent upstream primer and downstream primer, respectively.

**Table 2:** ALV reference strains used in this study.

Strains	Subgroup	Year	Country	Accession Number
MAV-1	A	1993	France	L10922
MQNCSU	A	2006	USA	DQ365814
PDRC-1039	A/E	2007	USA	EU070900
PDRC-3246	A/E	2007	USA	EU070901
PDRC-3249	A/E	2007	USA	EU070902
SDAU09C1	A	2009	China	HM452339
RAV-2	B	1986	USA	M14902
R-S-R B	B	1998	USA	AF052428
Prague C	C	1977	USA	J02342
R-S-R D	D	1992	Japan	D10652
RAV-0	E	1985	USA	M12172
ev-1	E	2000	USA	AY013303
ev-3	E	2001	USA	AY013304
SD0501	E	2007	China	EF467236
HPRS-103	J	1995	UK	Z46390
NX0101	J	2005	China	AY897227
SCAU-HN06	J	2006	China	HQ900844
TW-3593	*	2011	China	HM582658
Sp-53	*	2012	Japan	AB617820
Oki_009	*	2012	Japan	AB669433
Km_5844	*	2012	Japan	AB670312
Km_5943	*	2012	Japan	AB669897
Km_5845	*	2012	Japan	AB670314
JS13-DX5	K	2014	China	KF999961
JS13-LH1	K	2014	China	KF999962
JS11C1	K	2014	China	KF746200
GDFX0601	K/E	2014	China	KP686142
GDFX0602	K/E	2014	China	KP686143
GDFX0603	K/E	2014	China	KP686144
GD150509	K/E	2015	China	—
GD160403	K/E	2016	China	—
GD160607	K/E	2016	China	—

* The isolates whose subgroups are not known; “—” means that it has not been published in NCBI.

**Table 3:** Homology of nucleotide sequences of the long terminal repeat (LTR), gp85, and gp37 genes between the 3 isolates and other reference strains.

ALV Strains	GDFX0601			GDFX0602			GDFX0603		
	gp85	gp37	LTR	gp85	gp37	LTR	gp85	gp37	LTR
MQNSU (A)	85.1	94.4	68.1	85.2	94.6	67.8	84.9	94.7	67.4
R-S-RB (B)	83.2	93.6	59.0	83.2	93.8	59.5	82.9	93.9	59.0
Prague C (C)	88.4	93.7	70.0	88.5	93.9	70.3	88.2	94.1	70.0
R-S-RD (D)	78.2	94.3	57.2	78.4	94.4	57.7	78.1	94.6	57.2
HPRS-103 (J)	51.5	58.7	67.0	51.7	59.0	67.4	51.5	59.2	67.0
ev-1 (E)	87.8	98.7	98.2	87.9	98.9	98.5	87.7	99.0	98.2
ev-3 (E)	87.7	98.7	98.5	87.8	98.9	98.9	87.6	99.0	98.5
SD0501 (E)	87.8	98.4	96.5	87.9	98.5	96.9	87.6	98.7	96.5
TW3593 (*)	95.8	99.2	99.6	95.9	99.3	100	95.6	99.5	99.6
PDRC-1039 (A/E)	86.0	97.9	98.9	86.1	98.0	99.3	86.1	98.2	98.9
PDRC-3246 (A/E)	86.4	97.7	97.8	86.5	97.9	98.2	86.5	98.0	97.8
PDRC-3249 (A/E)	87.6	98.2	98.2	87.7	98.4	98.5	87.5	98.5	98.2
JS11C1 (K)	96.0	95.7	71.1	96.1	95.9	71.4	95.8	96.1	71.1
Km_5844 (*)	98.9	96.7	59.0	99.0	96.9	59.5	98.7	97.0	59.0
Sp_53 (*)	94.7	94.1	64.8	94.8	94.2	65.2	94.5	94.1	64.8
JS13-DX5 (K)	99.1	-	-	99.2	-	-	98.9	-	-

Note: The letters in () represent the virus subgroup; * The isolates whose subgroups are not known; - indicates no result could be obtained.

**Table 4:** The influence of ALV-K infection on mortality.

Mortality Rate *	Control	Group II	Group III
7 d	0%	0%	0%
14 d	0%	0%	3%
21 d	0%	0%	3.4%
28 d	0%	0%	3.6%
35 d	0%	0%	0%

* Mortality rate followed by different time.
